# Evaluation of lipid oxidative stress status and inflammation in atopic ocular surface disease

**Published:** 2010-11-19

**Authors:** Tais H. Wakamatsu, Murat Dogru, Igarashi Ayako, Yoji Takano, Yukihiro Matsumoto, Osama M.A. Ibrahim, Naoko Okada, Yoshiyuki Satake, Kazumi Fukagawa, Jun Shimazaki, Kazuo Tsubota, Hiroshi Fujishima

**Affiliations:** 1 Johnson & Johnson Ocular Surface and Visual Optics Department, Keio University School of Medicine, Tokyo, Japan; 2 Department of Ophthalmology, Keio University School of Medicine, Tokyo, Japan; 3 Department of Ophthalmology, Tokyo Dental College, Chiba, Japan; 4 Department of Ophthalmology, Kitasato Institute Hospital, Tokyo, Japan; 5 Department of Ophthalmology, Saiseikai Central Hospital, Tokyo, Japan

## Abstract

**Background:**

Although the oxidative stress status in atopic skin disease has been reported to be elevated, there are still no studies related to the status of oxidative stress in atopic ocular surface disease. The purpose of this study was to evaluate the ocular surface lipid oxidative stress status and inflammation in atopic keratoconjunctivitis (AKC) patients and normal subjects.

**Methods:**

Twenty eight eyes of 14 patients (9 males, 5 females) with AKC and 18 eyes of 9 age and sex matched (4 males and 5 females) normal healthy controls were examined in this prospective study. The severity of atopic dermatitis (AD) was scored by the SCORing Atopic Dermatitis (SCORAD) index. All subjects underwent Schirmer test, tear film break up time (BUT), fluorescein/Rose Bengal stainings, tear collection, and brush cytology from the upper palpebral conjunctiva. The brush cytology samples were stained with Diff-Quik for differentiation of inflammatory cells and immunohistochemistry (IHC) staining with HEL (hexanoyl-lysine) and 4-HNE (4-hydroxy-2-nonenal) to study lipid oxidation. HEL and cytokine (interleukin-4 (IL-4), interleukin-5 (IL-5), interleukin-10 (IL-10), tumor necrosis factor-alpha (TNF-α), interferon-gamma (IFN-γ)) levels were measured by enzyme-linked immunosorbent assay (ELISA) from tear samples of AKC patients and control subjects. Toluidine Blue and IHC staining with HEL, 4-HNE and cluster of differentiation 45 (CD45) were performed on papillary samples of AKC patients. This study was conducted in compliance with the “Declaration of Helsinki.”

**Results:**

The tear stability and vital staining scores were significantly worse in eyes of AKC patients (p<0.05) compared to the controls. Inflammatory cells and positively stained conjunctival epithelial cells for HEL and 4-HNE showed a significant elevation in brush cytology samples of AKC patients. Significantly higher levels of HEL and cytokines were detected in tears of AKC patients compared to controls. Papillary specimens also revealed many CD45 inflammatory cells as well as many cells positively stained with HEL and 4-HNE in IHC. A strong significant linear positive correlation between conjunctival inflammation and epithelial lipid oxidative stress status was observed. Conjunctival lipid oxidative stress also correlated strongly with tear HEL levels and epithelial damage scores.

**Conclusions:**

The ocular surface disease in AKC was characterized by marked tear instability, ocular surface epithelial damage, increase in inflammatory infiltrates and presence of increased lipid oxidation.

## Introduction

Atopic dermatitis (AD) is a worldwide public health problem with an increase in prevalence and morbidity in recent decades [1,2].

Atopic Keratoconjunctivitis (AKC) is a bilateral, chronic inflammation of the conjunctiva and lids associated with AD. The primary symptoms and signs of AKC include itching of the lid skin, periorbital area, conjunctiva, tearing, mucous discharge, burning, photophobia and blurred vision. The conjunctival involvement may vary from a normal looking conjunctiva to severe papillary hypertrophy, subepithelial fibrosis, and symblepharon formation. However, it is the corneal problems that are the vision-limiting factors [3]. Although the mechanisms involved in the allergic inflammation of AKC are not completed clear, T-cell driven inflammatory processes, infiltrations with mast cells, eosinophils and other inflammatory cells seem to play a major role. A better understanding of the mechanisms that underline the AKC is therefore critical for the design of a new and more effective treatment protocols for this disease.

Skin inflammation in AD is histologically characterized by intense infiltration of lymphocytes, monocytes and eosinophils which have been reported to release proinflammatory cytokines and reactive oxygen species (ROS) such as O_2_^-^, H_2_O_2_ and peroxinitrite (ONOO^-^), upon immunological and non-immunological stimulation [4-7].

Although the oxidative stress status in atopic skin disease has been reported to be elevated [8,9], there are still no studies related to the status of oxidative stress in atopic ocular surface disease. We investigated whether oxidative stress related changes were present in the ocular surface of patients with AKC comparing the results with healthy control subjects.

## Methods

### Subjects

Twenty-eight eyes of 14 AKC patients with AD (9 males, 5 females) aged between 11 and 43 years (mean: 22.6 years) as well as 18 eyes of 9 normal subjects aged from 20 to 35 years (mean: 25.2 years; 4 males and 5 females) were recruited from the Ocular Allergy Subspecialty Clinic of the Department of Ophthalmology, Mita Hospital, International University of Health and Welfare in this prospective study.

All AKC patients had active ocular disease in this study. The severity of AD was assessed by the Scoring Atopic Dermatitis (SCORAD) index [10,11]. Venous peripheral blood was collected and enzyme-linked immunosorbent assay (ELISA) test to detect the specific immunoglobulin E (IgE) antibodies to 26 allergens was performed using the MAST 26 Allergen Kit in all subjects (SRL, Tokyo, Japan). None of the patients had a history of Stevens-Johnson syndrome, chemical, thermal, radiation injury, bacterial, viral or toxic conjunctivitis; or underwent any ocular surgery that would create an ocular surface problem. As ethic board committee did not allow a washout period in subjects with an active disease to study the naïve ocular surface status, only patients who were recalcitrant to the same treatment regimen prescribed at their referral centers were recruited. No patient was being treated with systemic cytotoxic immunosuppressants, steroids, or prostaglandin inhibitors. Control subjects did not have any history of ocular or systemic disease and any history of drug or contact lens use that would alter the ocular surface as well. An informed consent about the procedures as well as permission from the Ethical Committee of Mita Hospital, International University of Health and Welfare was obtained. This study adhered to the tenets of the Declaration of Helsinki.

### Tear function tests and ocular surface vital staining scores

At slit-lamp examination, particular attention was paid to lid margins, tarsal and bulbar conjunctiva and cornea. Conjunctival injection grade was classified with a scale of severity score ranging from zero (no conjunctival injection) to 3 (severe conjunctival injection).

The standard tear film break up time (BUT) measurement was performed. Two microlitres of preservative-free combination of 1% fluorescein dye was instilled in the conjunctival sac. The dye was introduced to the conjunctival sac with a micropipette. The subjects were then instructed to blink several times for a few seconds to ensure adequate mixing of the dye. The interval between the last complete blink and the appearance of the first corneal black spot in the stained tear film was measured three times and the mean value of the measurements was calculated. A BUT<10 s was considered abnormal [12]. Fluorescein staining of the cornea was also noted and scored. Fluorescein staining scores ranged between zero and nine points [13]. A score >3 points was regarded as abnormal [14]. For further evaluation of tears, the standard Schirmer test with topical anesthesia (0.4% oxybuprocaine chloride) was performed. The standardized strips of filter paper (Showa Yakuhin Kako Co., ltd, Tokyo, Japan) were placed in the lateral canthus away from the cornea and left in place for 5 min with the eyes closed. Readings were reported in millimeters of wetting for 5 min. A reading of <5 mm was referred to as dry eye [15].

### Tear collection

Using a capillary micropipette, tears were gently collected from the external canthus, taking precaution to avoid reflex tearing. Tear samples were collected before the vital staining examination. Following collection, tears were placed in Eppendorff tubes and centrifuged at 13,600× g for 5 min at 4 °C. The supernatants were then stored at −80 °C until assayed.

### Conjunctival brush cytology

The brush cytology specimens were obtained after administration of topical anesthesia with 0.4% oxybuprocaine. Two adjacent nonoverlapping areas of central upper palpebral conjunctiva were used for sampling. Conjunctiva was scraped seven times with the Cytobrush-S (Medscand AB, Malmö, Sweden), the examiner holding the brush 2 cm away from the brush end, applying a gentle pressure to the conjunctiva. After sampling, the brushes were immediately placed in 1 ml of Hank’s buffered solution, and the containers were shaken to detach the cells from the brush. The suspended cells were collected using the Millipore filter technique employing filters with 8 μm pore size. One slide was allocated for the assessment of conjunctival inflammatory cells number by Diff Quik staining.

### Surgical technique for papillae resection and histopathological assessment of specimens

After topical anesthesia with 4% lidocaine eye drops, 0.2 ml of 2% lidocaine was injected to the upper fornix. The cobblestone-like papillae including subconjunctival connective tissues were removed using sharp dissection. Then, 0.1% methylprednisolone ointment was applied to the conjunctival sac, and a pressure eye patch was maintained for 1 day. Tissue samples were fixed overnight in formalin solution and processed for paraffin embedding. Sections (5 µm) were cut from paraffin wax blocks and mounted on pre-coated glass slides. Inflammatory cells were detected by toluidine blue staining and immunohistochemistry with anti-cluster of differentiation 45 (anti-CD45) primary antibody. Lipid oxidative stress status was visualized by immunohistochemistry with anti-HEL (anti- hexanoyl-lysine) and anti-4-HNE (anti-4-hydroxy-2-nonenal) primary antibodies.

### Immunohistochemical staining

Oxidative stress induced lipid peroxidation was assessed by immunohistochemical detection of HEL (hexanoyl-lysine; early phase oxidative stress marker) and 4-HNE (4-hydroxy-2-nonenal; late phase oxidative stress marker) protein adducts. The avidin–biotin–peroxidase complex (ABC) method was used for immunostaining. Brush cytology samples were fixed in paraformaldehyde 4% before the staining procedure. Tissue samples were fixed overnight in formalin solution and processed for paraffin embedding. Sections (5 µm) were cut from paraffin wax blocks, mounted on pre-coated glass slides, deparaffinized, and rehydrated. Antigen retrieval was achieved by microwaving in 10 mmol/l sodium citrate buffer for 5 min then cooling for 20 min. To block nonspecific background staining, sections were treated with normal horse serum (Vector Laboratories, Burlingame, CA) for 1 h at room temperature. The sections were then incubated for 90 min with the following monoclonal mouse primary antibodies: anti-4-HNE at a dilution of 1:4 (JaICA, Shizuoka, Japan), anti-HEL at a dilution of 1:10 (JaICA) and anti-CD45 at a dilution of 1:50 (BioLegend, San Diego, CA). For the negative controls, the primary antibody was replaced with mouse immunoglubulin G1 kappa (IgG1κ) (Sigma, St. Louis, MO). Endogenous peroxidase activity was blocked using 3.0% H_2_O_2_ in methanol for 3 min. The sections were incubated for 30 min with biotin-labeled horse anti-mouse IgG serum (Vector Laboratories, Burlingame, CA), followed by avidin-biotin-alkaline phosphatase complex (Vector Laboratories) for 30 min. The sections were washed in PBS buffer, developed in prepared 3,3′-diaminobenzidine (DAB) chromogen solution, lightly counterstained with hematoxylin, dehydrated, and mounted.

### Quantification of oxidative damage and eosinophil staining

All brush cytology specimens from each patient and control subjects were evaluated using light microscopy at 40× magnification for the presence of positive immunohistochemical staining for oxidative stress markers (HEL and 4-HNE) and eosinophils. The number of positively HEL and 4-HNE stained cells was counted within a total of one hundred conjunctival epithelial cells, in a masked fashion. The number of eosinophils was counted within a total of one hundred inflammatory cells. The counting technique was repeated 5 times in nonoverlapping random areas for each specimen and cell density results were expressed in percentage of stained cells.

### Enzyme-linked immunosorbent assay (ELISA) for tear HEL and Cytokine assessment

#### Measurement of tear HEL levels

A commercially available HEL enzyme-linked absorbant assay (ELISA; JaICA) was used to determine the tear HEL concentration, as reported previously [16]. We also investigated the correlation between tear HEL levels and numbers of cells positively stained with HEL in conjunctival brush cytology specimens.

#### Measurement of tear cytokine levels

Tear samples were taken from each eye with 10 µl glass capillary tubes in the same manner as described above. Following collection, the tears were centrifuged at 13,600× g for 5 min at 4 °C. The supernatants were removed and stored at −80 °C. The BD Cytometric Bead Array Human Th1/Th2 Cytokine Kit (BD Biosciences, San Jose, CA) was used in combination with the BD FACSCalibur™ flow cytometer (BD Biosciences) to qualitatively measure interleukin-4 (IL-4), interleukin-5 (IL-5), interleukin-10 (IL-10), tumor necrosis factor-α (TNF-α), and interferon-γ (IFN-γ) according to the manufacturer’s instructions, as previously reported [17,18]. The data acquired from the flow cytometer was processed using the BD™ CBA Analysis Software (BD Biosciences).

### Statistical analysis

Data were processed using Instat, GraphPad software version of InStat 3.0 (San Diego, CA). The Mann–Whitney test was used to compare the parameters between the AKC subjects and the normal controls. The correlation between tear HEL levels and number of cells positively stained with lipid oxidative stress markers in conjunctival brush cytology specimens as well as the correlation between corneal damage scores and number of cells positively stained with lipid oxidative stress markers and inflammatory cells were studied by using the Spearman correlation analysis. A probability level of less than 5% was considered statistically significant.

## Results

### Patient characteristics

Patients with AKC had active mild to severe AD with positive personal or family history of atopic disease. The mean SCORAD index score was 38.94±18.48. The most frequent sensitizing allergens in patients with AKC were *Dermatophagoides pteronyssinus*, cedar tree pollen, and *Phleum pratense* pollen. All patients had active AKC as evidenced by conjunctival injection, chemosis, papillary hypertrophy, tearing, and mucus discharge. There were no age- or gender-related differences between the patients and the control subjects. All patients complained of allergic and dry eye symptomatology, including itchiness, redness, grittiness, tiredness, discomfort and irritation. The mean conjunctival injection grade for AKC patients was significantly (2.0±1.0) greater than the normal control subjects (0.1±0.2; p<0.0001).

### Tear function tests and ocular surface findings

The mean BUT score in eyes with AKC was significantly lower than in eyes of healthy control subjects as shown in Table 1 (p<0.05). The mean BUT values were 5.1±2.6 s and 7.8±2.3 s in AKC patients and control subjects, respectively. The mean Schirmer test scores were normal in both patient and control groups with a significantly lower value in eyes with AKC compared with eyes of control subjects (p<0.05), (Table 1). The mean fluorescein scores were 3.9±3.2 points and 0.4±1.0 points in AKC patients and control subjects, respectively. The differences were statistically significant (p<0.0001).

**Table 1:** Comparison of tear functions and ocular surface vital stainings between AKC patients and healthy control subjects.

**Tear functions and ocular surface vital staining scores**	**AKC patients**	**Control subjects**
Tear break-up time (BUT - seconds)	5.1±2.6*	7.8±2.3
Schirmer test 1 (mm)	11.7±8.7*	18.1±8.1
Fluorescein staining (0-9 points)	3.9±3.2*	0.4±1.0
Conjunctival injection grade (0-3points)	2.0±1.0*	0.1±0.2

The asterisk indicates a p< 0.05 using the Mann-Whitney Test.

### Staining and quantification for eosinophils in brush cytology samples

**Figure 1:**
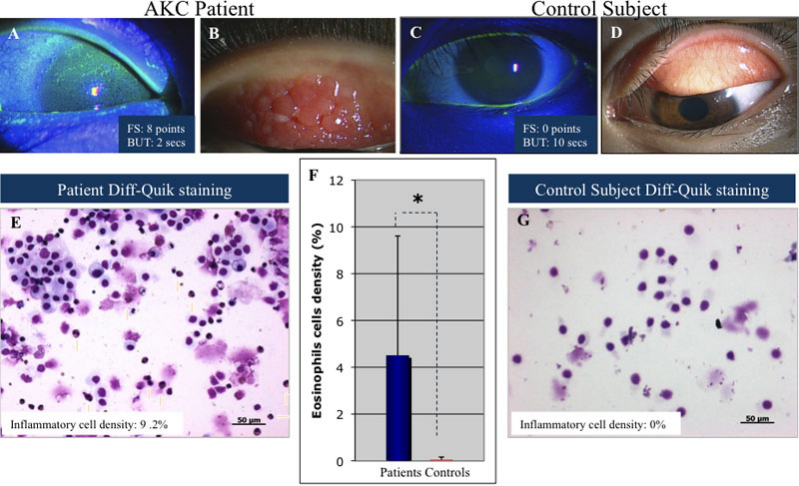
Representative anterior segment photographs and brush cytology samples showing comparison of inflammatory cell infiltrates in an AKC patient and a control. Anterior segment photographs show extensive corneal damage visualized by the fluorescein staining in patient with AKC. Note that the superficial punctate keratopathy is present in almost all the surface of the cornea and is associated to the increased proliferation of the conjunctival papillae (**A**, **B**). The photograph on the left side represents a normal cornea with no conjunctival proliferation on the tarsal conjunctiva (**C**, **D**). Note the extensive inflammatory infiltrates in Diff-Quik staining from brush cytology samples in the AKC patient (**E**) compared with the healthy control subject (**G**). The graphic shows the comparison of mean percentage inflammatory cells stained by DQ between the two groups (**F**).

Brush cytology reveled significantly higher percentage of eosinophils in eyes with AKC (4.5±5.12) compared with healthy control eyes (0.05±0.11) as shown in Figure 1. (p<0.0001). The number of eosinophilic cells showed a significant positive correlation with corneal fluorescein staining scores (data not shown; r=0.62, p<0.0001).

### Immunohistochemistry stainings for HEL and 4-HNE and quantification of lipid oxidative stress damage

**Figure 2:**
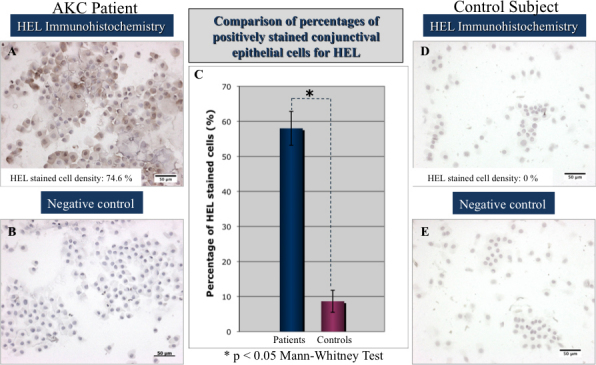
Representative immunohistochemistry stainings for the early lipid oxidation marker in brush cytology specimens from an AKC patient and a control subject. Note the extensive lipid oxidative stress damage in the HEL immunohistochemistry staining from brush cytology samples of an AKC patient (**A**) compared to the healthy control subject (**D**). Note the significantly higher percentage of cells stained by HEL in patients with AKC (**C**). **B** and **E** represent the negative controls from the immunohistochemistry.

**Figure 3:**
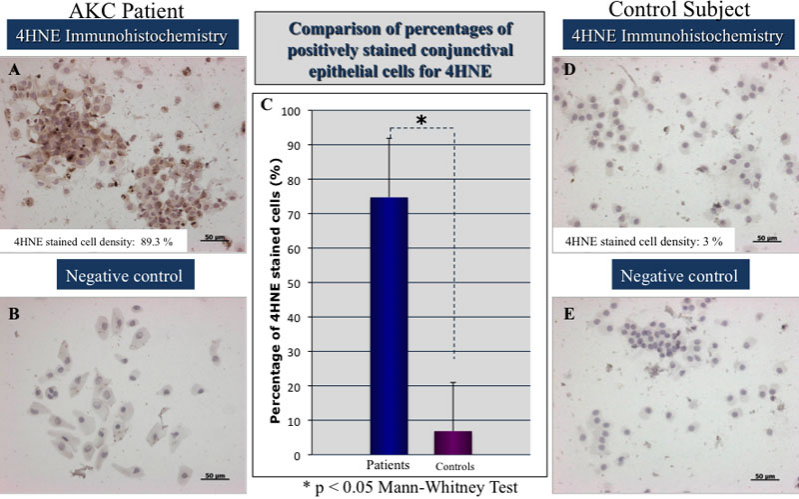
Representative immunohistochemistry stainings for the late lipid oxidation marker in brush cytology specimens from an AKC patient and a control subject. Note the extensive lipid oxidative stress damage in the 4HNE immunohistochemistry staining from brush cytology samples of an AKC (**A**) patient compared to the healthy control subject (**D**). Note the significantly higher percentage of cells stained by 4HNE in patients with AKC (**C**). **B** and **E** represent the negative control from the immunohistochemistry.

All conjunctival specimens from AKC patients revealed marked staining for HEL and 4-HNE, in the epithelial cells from brush cytology specimens compared to control subjects as shown in Figure 2 and Figure 3. The average percentages of positively stained epithelial cells for 4-HNE and HEL antibodies in the AKC subjects were 74.71±17.15% and 58.01±21.61%, respectively. In the control group, the average percentages of positively stained conjunctival epithelial cells for 4-HNE and HEL were 6.85±14.13%, and 8.62±13.3%, respectively. The differences were statistically significant (p<0.0001).

### ELISA for measurement of tear cytokine levels

**Figure 4:**
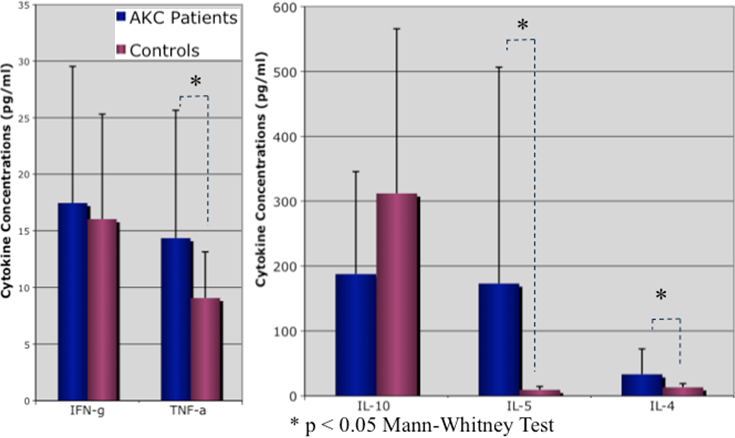
Comparison of tear cytokine levels between AKC patients and age and sex matched healthy control subjects.

IL-4, IL-5, and TNF-α cytokine concentrations were significantly higher in the eyes of patients with AKC compared with the eyes of healthy control subjects, as shown in Figure 4 (p<0.05). The mean IL-4, IL-5, and TNF-α tear cytokine levels were: 31.01±37.95 pg/ml, 173.09±333.5 pg/ml and 14.35±11.31 pg/ml in AKC patients, respectively. The corresponding tear cytokine levels in control subjects were: 15.88±9.59 pg/ml, 9.36±5.16 pg/ml, 9.05±4.06 pg/ml, respectively. Tear IL-5 and TNF- α cytokine concentration showed a significant linear positive correlation with conjunctival cell numbers positively stained with HEL. Tear IL-5 cytokine also showed a significant linear positive correlation with conjunctival cell numbers positively stained with 4-HNE as shown in Table 2.

**Table 2:** Correlations between tear IL-4, IL-5, and TNF-α cytokine levels and HEL and 4-HNE positively stained cells.

** **	**Oxidative lipid peroxidation marker staining (%)**			
** **	**HEL**		**4-HNE**	
**Protein**	**Spearman correlation coefficient**	**p value**	**Spearman correlation coefficient**	**p value**
IL-4	r=0.2070	>0.05	r=0.0820	>0.05
IL-5	r=0.7806	<0.0001*	r=0.5646	<0.05*
TNF-α	r=0.4148	<0.05*	r=0.2751	>0.05

### ELISA for measurement of tear HEL levels

**Figure 5:**
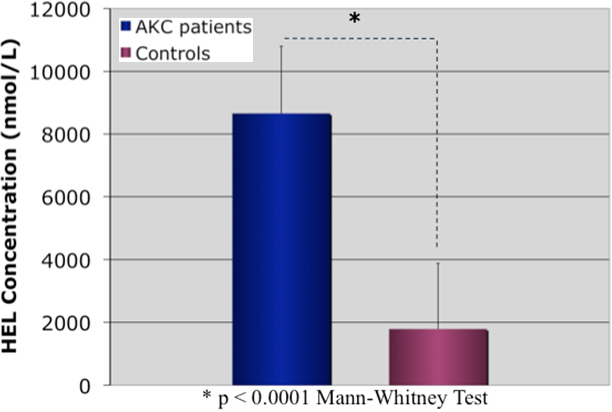
Comparison of tear hexanoyl-lysine levels between AKC patients and age and sex matched healthy control subjects.

The tear HEL concentration was significantly higher in the eyes of patients with AKC (8653.1±2145.7 pg/ml) compared with the eyes of healthy control subjects (1789.4±2089.8 pg/ml), as shown in Figure 5 (p<0.0001).

### Immunohistochemistry staining for conjunctival papillae samples

**Figure 6:**
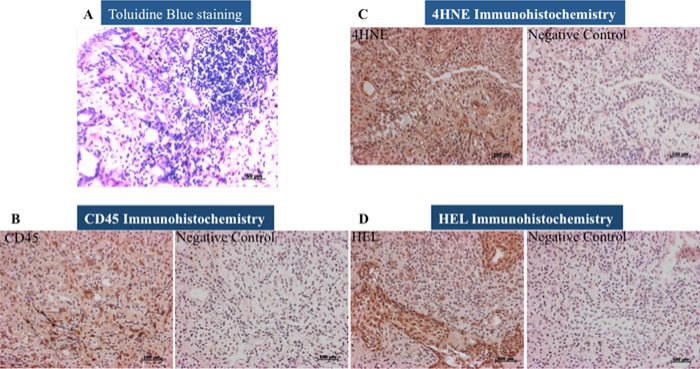
Representative immunohistochemistry staining for oxidative stress and inflammatory cell markers in papillae samples from an AKC patient. Note the presence of several inflammatory cells characterized by eosinophils in dark-pink color stained by toluidine blue (**A**). The polymorph inflammatory cells are stained by the anti-CD45 antibody and evidenced by dark-brown staining (**B**). The presence of oxidative stress damage is observed by the immunohistochemistry staining for 4HNE (**C**) and HEL (**D**). Note that the dark-brown color represents the oxidative stress damage sites.

Papillary tissues from AKC patients presented marked positive staining for CD45 antibody, oxidative stress damage markers (HEL and 4-HNE) and increased number of eosinophils, Figure 6.

### Correlation of Tear HEL concentration with conjunctival HEL staining

**Figure 7:**
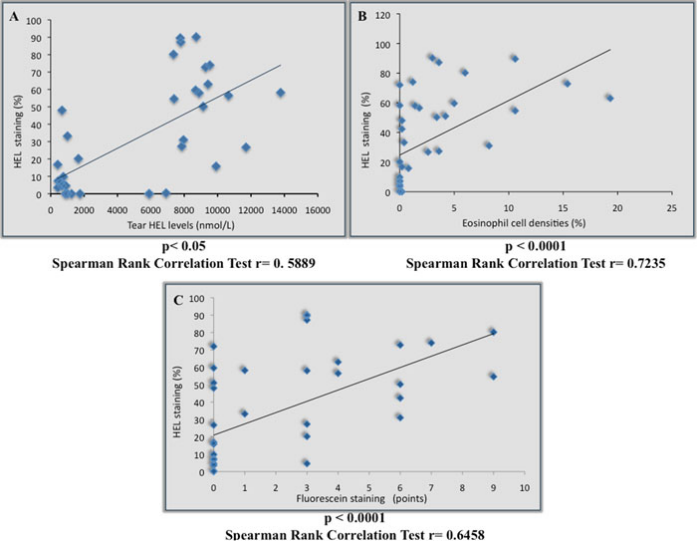
Correlation between inflammation, corneal epithelial cell damage and HEL tear levels. Conjunctival lipid oxidative stress was strongly correlated with tear HEL levels (**A**) and conjunctival inflammation (**B**). A significant linear positive correlation between epithelial damage scores and epithelial lipid oxidative stress status was observed (**C**).

The tear HEL concentrations showed a significant linear positive correlation with percentages of positively stained cells for HEL in conjunctival brush cytology specimens (r=0.59, p<0.05) as shown in Figure 7A.

### Correlation of eosinophil cell density and corneal epithelial damage with conjunctival HEL staining

The percentages of positively stained cells for HEL showed a significant linear positive correlation with corneal fluorescein staining scores (r=0.65, p<0.0001) and with the eosinophil cell numbers (r=0.72, p<0.0001) as shown in Figures 7B,C.

### Correlation of eosinophil cell density and corneal epithelial damage with conjunctival 4HNE staining

**Figure 8:**
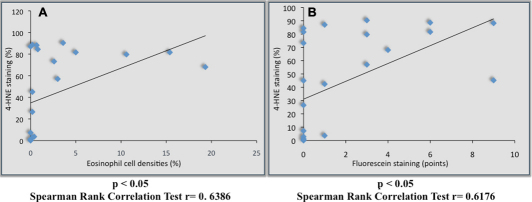
Correlation between inflammation, corneal epithelial cell damage and 4-HNE staining. Conjunctival lipid oxidative stress (4-HNE) was correlated with conjunctival inflammation (**A**) and corneal epithelial damage scores (**B**).

The percentages of positively stained cells for 4-HNE showed a significant linear positive correlation with corneal fluorescein staining scores (r=0.64, p<0.05) and with the eosinophil cell numbers (r=0.62, p<0.05) as shown in Figures 8A,B.

## Discussion

Cells generate energy by reducing oxygen to water during which reactive oxygen species are generated. The generation of free radicals has been reported to lead to tissue injury through induction of inflammation in several disease states including sepsis, ischemic heart and brain disease, atherosclerosis, asthma and AD [8,9,19-23].

Increased oxidative stress status, specially elevation of ROS, has been described in patients with AD. Indeed, urine concentrations of 8-hydroxy-2’-deoxyguanosine (8-OHdG), a marker for oxidative DNA damage, and acrolein-lysine adducts, a marker of lipid peroxidation, have been reported to be higher in AD patients compared to controls [8,9]. Oxidative stress changes in AD have been reported extensively in Dermatology literature. Niwa et al. confirmed the presence of lipid peroxidation, detected by 4- HNE, in the stratum corneum of patients with AD [24]. The skin inflammation in AD was histologically characterized by intense infiltration of lymphocytes, monocytes and eosinophils [4,25]; all of which may release bioactive substances including inflammatory cytokines and ROS upon immunological and non-immunological stimulation [5]. There is futher evidence in the literature that circulating polymorphonuclear leucocytes from AD patients are hyperreactive, showing enhanced release of reactive oxygen intermediates [26,27], suggesting the possibility that these changes may lead to a vicious cycle further causing immune activation, oxidative damage, and propagation of skin inflammation.

In the field of Ophthalmology, oxidative stress damage has been reported to play a role in several ocular diseases including age-related macular degeneration [28,29], cataract [30], uveitis [31], retinopathy of prematurity [32], corneal inflammation [33,34], keratitis [35], and conjunctivochalasis [36], an aging disease of the conjunctiva characterized by conjunctival laxity.

Although the oxidative stress status in atopic skin disease has been reported to be elevated, there are still no studies related to the status of oxidative stress in atopic ocular surface disease.

In this study, we investigated the lipid oxidative stress related changes in the tear film and brush cytology samples of patients with AD comparing the results with healthy control subjects. We also performed a histopathological assessment of lipid peroxidation staining in papillary resection samples of patients with severe AKC.

The tear stability, quantity, ocular surface epithelial damage and conjunctival injection scores were significantly worse in patients with AKC compared with controls which were consistent with previously published studies. Brush cytology revealed a significantly higher extent of inflammatory infiltrates, mainly of eosinophils in AKC patients. Another piece of evidence for increased ocular surface inflammation was based on significant increases in the concentration of TNF-α, IL-5, and IL-4 in tears of patients with AKC compared with controls. Not only the systemic disease process that favors a Th2 cytokine dominant response in AD but the environmental conjunctival allergen exposure might have led to the recruitment and activation of inflammatory cells in the conjunctiva.

Allergen exposure has been shown to be associated with release of IL-5 and IL-13 from inflammatory cells in allergic airway exposure and associated with oxidative stress in BALB/c mice [37]. In vitro studies on primary cultures of purified human CD4^+^ T cells showed evidence that oxidative stress could be induced which in turn modulated T cell polarization toward Th2 cytokine producing cells, including IL-4, IL-5, and IL-13 [38]. Inflammatory cells are also known to release reactive oxygen products. It is our belief that allergens induce recruitment of inflammatory cells including mast cells, lymphocytes, eosinophils, and neutrophils in AKC, which not only release inflammatory cytokines but free oxygen radicals as well.

Our efforts to investigate the lipid oxidative stress changes interestingly revealed higher percentages of cells positively stained for HEL (early phase lipid peroxidation marker) and 4-HNE (late phase lipid peroxidation marker) in palpebral conjunctival samples suggesting an increased lipid peroxidation status in AKC. The lipid peroxidation marker HEL was also significantly increased in tears of patients with AKC compared with controls.

Although palpebral conjunctival tissue samples from healthy control subjects could not be obtained due to lack of ethic board permission, palpebral conjunctiva covering the papillary formations during papillary resection surgery in patients with severe AKC showed dense mast cells and CD45^+^ inflammatory cells as well as marked stainings for HEL and 4-HNE antibodies providing further evidence that conjunctival inflammation and lipid peroxidation coexisted in AKC.

There is increasing evidence that atopic diseases are intimately linked to the generation of oxidative stress. While the oxidative stress could be the consequence of oxygen radical production by macrophages, neutrophils, and eosinophils, it is important to understand that oxidative stress could also be an inciting factor in the generation of ocular surface inflammation. Oxidative free radicals directly oxidize various macromolecules including lipids. It has been described in the literature that lipid peroxides and their break-down products, such as HEL and 4-HNE, can directly or indirectly affect many functions integral to cellular and organ homeostasis. As a result, the increased membrane lipid peroxidation may evoke and/or increase the immune and inflammatory response, activate gene expression and cell proliferation, or initiate apoptosis [39]. Thus, a close relationship between ROS production, peroxidative lipid membrane damage and an inflammatory pathological process may be postulated for the ocular surface disease in AKC.

Our correlation analysis showed a significant positive correlation between tear HEL levels and conjunctival HEL staining whereas another strong positive correlation was observed between eosinophilic inflammatory cell numbers and the extent of early and late lipid peroxidation as evidenced by the higher percentages of positively stained cells for HEL and 4-HNE in specimens harboring higher eosinophil densities. The extent of early and late lipid peroxidation marker stainings also showed a strong positive correlation with corneal epithelial damage.

Peroxidation of membrane lipids might have very well induced perturbation of ocular surface epithelial cellular functions causing breakdown and death of epithelial cells which might have contributed to increased epithelial fluorescein staining scores. TNF-α, which is known to induce inflammation and cell death, was also elevated in tears of AKC patients. The pathways regulating the elevation of lipid oxidation status and linking it to inflammation are still unclear. Whether upregulation of STAT 6 with increased NF-kB activity results in Th2 favored cytokine response and induce oxidative stress in allergic ocular surface disease remains to be investigated in future studies.

It also remains the further goal of future studies to determine short and long-term conjunctival epithelial cytology staining status with oxidative stress markers and their correlation to inflammatory cytokine markers that may open up some new avenues of categorizing, diagnosing, and treating different cryptic elements of AKC and AD. Further studies investigating the simultaneous alterations of the ocular surface anti-oxidant status will also provide invaluable information.

It is also our wish that the observations from the current study will pave the way to newer studies testing the efficacy of anti-oxidant treatment strategies in AKC. Indeed, antioxidant defense impairment in atopic dermatitis has been confirmed in a study reporting that N-acetyl cystein (NAC) was able to down-regulate Th2-secreted-cytokines, such as IL-4, IL-5 with a subsequent over-activation of the Th1 response, suggesting that in Th2 related diseases, such as atopic dermatitis, NAC might serve as a possible therapeutic agent [40]. Recent studies also suggest squalene monohydroperoxide is a primary oxidized lipid generator which is elevated in atopic dermatitis. Antioxidants, such as catechin, or ascorbic acid which have been shown to markedly decrease the cytotoxicity of squalene monohydroperoxide may find roles in the treatment of AKC in the future [41].

In summary, this study provided the first evidence that lipid peroxidation and inflammation coexisted in the conjunctiva of patients with AKC, a finding which might hold the keys to future explanations for the pathogenesis of the ocular surface disease in AKC.
